# Parastomal Hernia Prevention Guidelines: Methodology and Limitations

**DOI:** 10.3389/jaws.2025.15202

**Published:** 2025-08-12

**Authors:** Stavros A. Antoniou

**Affiliations:** ^1^ Department of Surgery, Papageorgiou General Hospital, Thessaloniki, Greece; ^2^ Papageorgiou GRADE Center, Thessaloniki, Greece

**Keywords:** parastomal hernia, prevention, prophylaxis, guidelines, European hernia society

## Introduction

The European Hernia Society (EHS) Guidelines published in 2023 provide an updated summary of the best available evidence on the prevention of parastomal hernias [1]. In these guidelines, the panel provided a conditional recommendation for the use of mesh in patients with an end colostomy and a fair life expectancy, and a strong recommendation for the use of mesh in patients at high risk of developing a parastomal hernia, including those with a history of abdominal wall hernias, connective tissue disorders, obesity, or patients who are likely to be treated with chemotherapy. Previous guidelines from the same Society published in 2018 had issued a strong recommendation for all patients with an end colostomy [2].

Studies conducted around the time of the most recent guideline publications, examining the use of prophylactic mesh in Europe, indicate that while the proportion of surgeons employing prophylactic mesh is rising, adherence to the recommendations remains very low [3–5]. The reasons behind this have not yet been studied systematically and in depth.

## Summary of the Data

The evidence comprised 12 randomised controlled trials, 3 of which provided long-term follow-up data [6]. Summary analyses indicate:

• Prophylactic mesh and standard end colostomy carry a similar risk of major perioperative complications (Clavien-Dindo ≥3).
• Patients with prophylactic mesh have on average a 67% lower risk of a parastomal hernia in the long term. In other words, 32% of patients without mesh will develop a parastomal hernia, against 11% of patients with mesh.
• Patients with a prophylactic mesh have on average an 82% lower risk of surgery for a parastomal hernia compared to patients without mesh–or 2% versus 5%, respectively.
• There is no difference in quality of life.

## Looking Beyond the Data

These data come from randomised trials, which are known to provide rigorous assessments of postoperative outcomes [7]. Some of these studies have used imaging studies to assess the presence of a parastomal hernia. The results of studies using clinical, or a combination of clinical and radiological assessments did not differ. Furthermore, meta-regression analyses indicated a more pronounced effect with an increased duration of follow-up [8]. In other words, prophylactic mesh appears to prevent, but also delay the occurrence of parastomal hernias in patients who will develop them.

In clinical practice, surgeons who use a prophylactic mesh for both hernia prevention and the treatment of parastomal hernias, observe small recurrences that infrequently incur practical problems with abdominal wall function, aesthetics, or the application of stoma bags. This contrasts with the frequently encountered complicated bulgy parastomal hernias without prophylactic mesh. Reoperation in patients with a prophylactic mesh and parastomal hernia recurrence is a rather rare scenario, and when this is necessary, resection of the stoma does not extend beyond what would be resected when revising a typical colostomy.

## The Issue of Patient-Centred Surgery

A key feature of modern guidelines is that they bring the patient perspective to the forefront. Patient preferences are the primary factor that drives clinical practice [9]. In line with Grading of Recommendations, Assessment & Evaluation (GRADE) and Guidelines International Network (GIN) guideline development standards [10, 11], the most recent EHS guidelines involved a diverse panel of field surgeons, researchers, stoma care nurses, methodologists, systematic reviewers, statisticians and patient partners–specifically, patients with lived experience of colorectal resection with an end colostomy. The panel of surgeons, stoma care nurses and patient partners were presented with the evidence, and the recommendations were based on the patient perspective.

The panel was asked:

If you were a patient about to have an end colostomy, and you were informed that:

• you would have a 32% risk of developing a parastomal hernia without mesh and an 11% risk with mesh,
• you would have a 2% risk of surgery for a parastomal hernia with mesh, versus 5% without mesh,
• and you would have no differences in other key endpoints, which management option would you prefer?

If almost all patients opted for a prophylactic mesh, a strong recommendation would be warranted. However, if the majority of patients preferred a prophylactic mesh, but a non-negligible proportion did not, then a conditional recommendation suggests that management options should be discussed with the patient.

In the EHS parastomal hernia prevention guidelines, panel members unanimously agreed that there would be minimal variation in patient preferences; therefore, they supported a strong recommendation.

Nevertheless, we opted to provide a more conservative set of recommendations, specifically a strong recommendation for high-risk patients, and a conditional recommendation for average- and low-risk patients. The reasons are outlined below.

## Limitations of the Evidence

The body of evidence clearly shows the direction of the effects, despite single randomised trials and observational studies indicating no effect [12–15]. It is well known that individual studies are prone to type II statistical error (that is, false negative results, because the sample size is too small to detect an actual effect) [16]; they do not reflect variations in practice, but rather the practice of one or a few participating institutions [17]; and frequently have methodological limitations, especially detection bias, due to the higher intensity of and different patterns of follow-up in patients receiving the intervention [18, 19]. Furthermore, they typically include a subset of patients from the wider patient population, usually healthier patients with fewer risk factors [20, 21].

Similarly, previous meta-analyses have not applied rigorous methods to appraise the certainty of the evidence, such as risk of bias assessment at the outcome level, rather than at the study level, sensitivity analyses of studies at low versus higher risk of bias, and rating the certainty using decision anchors [22, 23].

Despite the fact that the evidence is sufficiently robust to support a strong recommendation even for low- or average-risk patients, there are conceptual barriers that prevent the wide implementation of these are summarized in the evidence-to-decision framework that accompanies the recommendations.

First, surgeons have limited experience in using a mesh at the stoma site. This issue becomes more complex if we consider that the most effective site for placing the mesh may be the retrorectus or retromuscular position.

Second, there are concerns among surgeons that the mesh may become infected. Despite robust evidence of similar risks of Clavien-Dindo class ≥3 complications, there is a culture of ‘resistance to change’ that makes it difficult to be persuasive in shifting long-held clinical practices [24].

Third, there is a growing movement against the use of mesh in several European countries, which has influenced the opinions of some patients [25]. Even if these concerns are not grounded in scientific evidence, patient preferences must still be acknowledged. Shared decision-making between the patient and the surgeon is essential to ensure informed consent regarding the operative approach.

## Discussion

Bridging the gap between evidence-informed and empirical surgical practice remains a significant challenge. Encouragingly, however, the new generation of surgeons is more open to questioning established dogmas and embracing change. However, several obstacles must still be addressed to achieve optimal surgical research and practice.

### Surgical Research Must Follow the Highest Methodological Standards

Trials and observational studies must provide data on key outcomes, such as major complications, rather than composite outcomes of overall complications. If the distribution of major and minor complications is skewed between the groups, the summary outcome will be misleading.

The outcome assessment of both observational and randomised studies must be blinded. This ensures an unbiased assessment of the outcome, both in terms of intensity and follow-up methods.

Furthermore, the patient perspective must be a key consideration when measuring outcomes. For example, although radiological assessment is informative, primarily from a research lens, clinical assessment is paramount from the patients’ perspective.

Long-term follow-up of key outcomes is essential for the objective evaluation of surgical effectiveness. Substantial evidence indicates that the prophylactic benefits of mesh become more apparent over time, and that small parastomal hernias may have a limited impact on patients’ wellbeing. In this context, the publication of short-term follow-up data may offer limited value, as previous short-term studies have often been inconclusive and, at times, potentially misleading.

### Surgical Practice Must Be Contextualised and Follow Research Evidence

Surgeons must be aware that as high as 41% of patients with an end colostomy will develop a parastomal hernia in the long term. This incidence is particularly high, and the beneficial effect of a prophylactic mesh is pronounced because of this high baseline risk.

Patients should be informed of the high risk of parastomal hernias in the absence of prophylactic measures, along with the significantly reduced risk associated with the use of a prophylactic mesh. The relevance of this risk can vary depending on the underlying disease and surgical indication. For example, a patient undergoing proctocolectomy for familial adenomatous polyposis may place considerable importance on minimising the risk of a parastomal hernia. In contrast, for an older patient with stage IV low rectal cancer, the long-term implications of a parastomal hernia may be less clinically significant. A key outcome to be measured in future studies is quality of life, which can be affected by factors that are not always well captured, such as the size of the parastomal hernia or difficulties in applying the stoma bag.

Surgical dogmas are evolving, albeit more slowly than warranted. Innovations, such as minimally invasive techniques, are driving this shift. The use of synthetic meshes is becoming more accepted even in clean-contaminated and contaminated fields [26]. History has shown that the delayed adoption of well-established research evidence can expose patients to unnecessary risks and complications [27]. Effective communication among surgeons–through conferences, clinical visits, fellowships, and educational courses–plays a critical role in promoting the dissemination and adoption of evidence-informed practices.
